# Prehabilitation: a narrative review focused on exercise therapy for the prevention of postoperative pulmonary complications following lung resection

**DOI:** 10.3389/fmed.2023.1196981

**Published:** 2023-10-02

**Authors:** Emre Sertaç Bingül, Nüzhet Mert Şentürk, Ata Murat Kaynar

**Affiliations:** ^1^Department of Anesthesiology, Istanbul Faculty of Medicine, Istanbul University, Istanbul, Türkiye; ^2^Department of Anesthesiology, Acibadem University School of Medicine, Istanbul, Türkiye; ^3^Department of Anesthesiology and Perioperative Medicine, University of Pittsburgh, Pittsburgh, PA, United States; ^4^The Center for Innovation in Pain Care (CIPC), University of Pittsburgh, Pittsburgh, PA, United States; ^5^Department of Critical Care Medicine, University of Pittsburgh, Pittsburgh, PA, United States; ^6^The Clinical Research, Investigation, and Systems Modeling of Acute Illness (CRISMA) Center, University of Pittsburgh, Pittsburgh, PA, United States

**Keywords:** prehabilitation, lung resection, thoracic anesthesia, thoracic surgery, enhanced recovery after surgery, physical fitness

## Abstract

Extensive preventive strategies in the perioperative period are popular worldwide. Novel “prehabilitation” approaches are being defined for every individual surgical discipline. With intention to reduce perioperative morbidity, “prehabilitation” was developed to increase “physical wellness” considering exercise capacity, nutritional status, and psychological support. Thus, prehabilitation could be well-suited for patients undergoing lung cancer surgery. Theoretically, improving physical condition may increase the chances of having a better post-operative course, especially among frail patients. In this review, we describe the concept of prehabilitation with possible benefits, its role in the Enhanced Recovery After Surgery protocols, and its potential for the future.

## Introduction

Major surgeries are associated with complications resulting in prolonged hospital stay, increased resource utilization, and decreased survival (1–3). Although different incidences have been reported, postoperative pulmonary complications (PPCs) are more common than postoperative cardiovascular events (4). According to the European Perioperative Clinical Outcome (EPCO) study group, the incidence of PPCs may reach up to 33%, and these complications include respiratory failure, atelectasis, pneumonia, pleural effusion, bronchospasm, and pneumothorax (3, 5, 6).

In the case of surgical resection of lung malignancies, the presence of PPC following lung surgery was a significant predictor of cancer recurrence (7, 8). It has been shown that, there is a four-times increased risk for recurrence in patients who experienced “pulmonary” complications after lung surgery. In addition, almost 60% of patients with lung cancer have chronic obstructive pulmonary disease (COPD) which increases the risk for PPC (9).

Breathing exercises and incentive spirometry are the most used approaches postoperatively with the intention of reducing the respiratory complications. However, existing data are still controversial, and quality of evidence is low. Studies suggest occasional recovery in pulmonary function tests (PFT), but the effect on PPCs is insignificant (10–13). Possible mechanism might be the low compliance of patients with such exercises, yet it is still advised to use incentive spirometry after surgery since it is cheap and practical (14, 15).

At this point, two other options may be considered. First, would a “pre”-rehabilitation be more beneficial and reduce PPCs? Second, would a different preoperative exercise modality change the outcomes? Until last decade, research suggested that postoperative rehabilitation did not prevent pulmonary complications despite enhanced physical fitness (16). However, with the increasing popularity of enhanced recovery after surgery (ERAS) programs, holistic rehabilitation concept has become prominent for major surgeries.

### Preoperative risk assessment

A thorough examination of the patients is essential to assess perioperative risk (3, 6, 17). To obtain a comprehensive understanding, physicians should evaluate the patients on individual basis. From the “classic” clinical judgment, American Society of Anesthesiologists Physical Status (ASA-PS), Revised Cardiac Risk Index (RCRI), Assessing Respiratory Risk in Surgical Patients in Catalonia (ARISCAT), and STOP-BANG scores are reliable tools for risk stratification providing a general organ function perspective. Among these, ARISCAT is a pragmatic scoring system that calculates the risk of PPC occurrence according to age, preoperative SpO_2_, history of acute respiratory infection, presence of preoperative anemia, surgical incision, surgical duration, and urgency of the surgery, which are also some of the independent risk factors for pulmonary complications (6). There are many more independent risk factors identified for carrying a risk for postoperative cardiorespiratory events, and one of them is the “functional status,” which is very popular due to its “modifiable” nature (18).

Functional status, which also refers to aerobic capacity, is an indicator of physical fitness and cardiothoracic relation. A good aerobic capacity suggests that the heart and lungs are working concordantly, and this is crucial for patients undergoing major surgery while trying to reduce postoperative complications. Several surrogates such as Metabolic Equivalent Tasks (METs), Duke Activity Status Index (DASI), 6-min walking distance, and stair climbing test are used in order to determine the functional status and identify the physically unfit patients, yet measuring the peak oxygen consumption (VO_2_max) via Cardiopulmonary Exercise Testing (CPET) is the gold standard (19, 20). DASI is a 12-item questionnaire which is already validated in patients with cardiopulmonary disease, and appears to be detecting actual METs accurately (21–24). Therefore, it may be chosen as a practical surrogate and useful alternative for CPET (20).

### Improved functional status improves aerobic capacity

Peak oxygen consumption of 15 ml/kg/min is considered as a cut-off value to define who is at high risk for major cardiovascular events, and VO_2_ max 10 ml/kg/min is generally the threshold for anaerobic metabolism pointing an excessively reduced capability of oxygen usage. Under practical terms, 15 mL/kg/min VO_2_ max refers to 4 METs, which generally equals to ability of climbing two flights of stairs, slow tempo walking, or light-to-moderate effort. Aerobic capacity is improvable with appropriate strategies (25). It has been shown that 1-MET increase in the physical capacity results in serious drop of all-cause and cardiovascular mortality (HR of 0.46–0.89) (26). Poor physical fitness is associated with increased postoperative morbidity and mortality and detecting the patients with high-risk help physicians to provide a time window to intervene and enhance outcomes. Modifiable risk factors such as frailty, nutritional status, sleep hygiene, anemia, alcohol consumption, and smoking should be revisited, and necessary corrections should be made accordingly which might eventually benefit on total hospitalization and costs (27).

**Figure 1:**
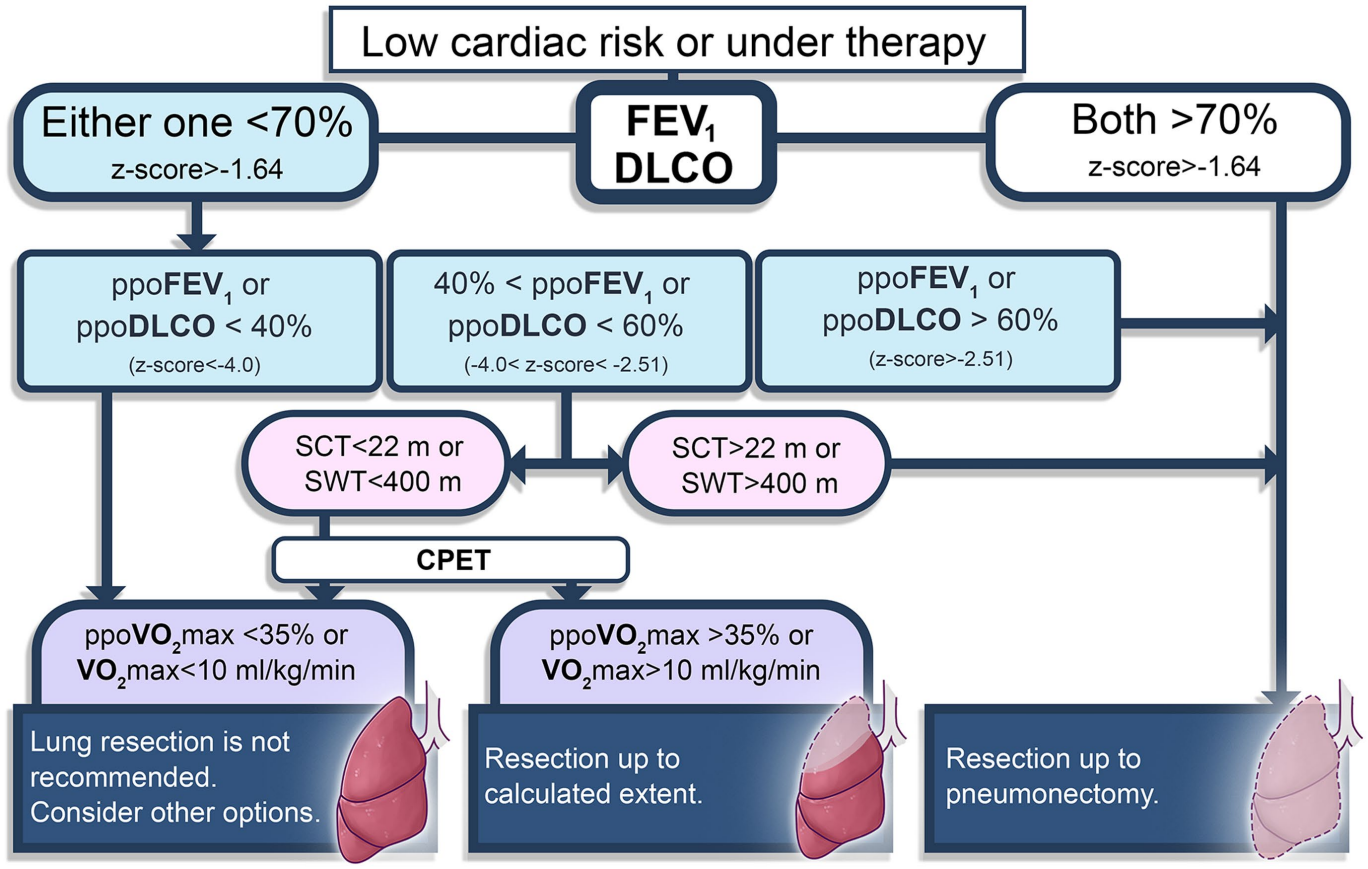
Brief diagram of preoperative evaluation for lung resection surgery patients (19, 30). FEV_1_, forced expiratory volume in one second; DLCO, diffusion capacity of the lung for carbon monoxide; ppo, predicted postoperative; SCT, stair climbing test; SWT, Shuttle-walk test; CPET, cardiopulmonary exercise test. Pulmonary function test parameters are also presented using European Respiratory Society recommendations (29).

Determining the physical capacity is also important on the decision-making process of the surgery. The extent of lung resection is evaluated based on spirometry, diffusion capacity of the lung for carbon monoxide (DLCO) and functional status. PFT results should be interpreted together with aerobic capacity, since good expiratory volume or total capacity might be accompanied with PPCs when the aerobic capacity is critically low (28). Predicted postoperative (ppo) values of forced expiratory volume in one second (FEV_1_) and DLCO higher than 60% (−1.64 > z-score > 2.51) represent a good lung condition that allows major resections up to pneumonectomy. However, if one of these values are between 40 and 60% of predicted (−2.51 > *z*-score > −4.0), then functional status evaluation should be performed (29). Simple tests such as shuttle-walk (>400 m) or stair-climbing (>22 m) may be chosen, and in case of uncertain results, CPET provides the actual VO_2_ max. Current functional status helps to decide of how large the resection can be. Patients with lower than 10 mL/kg/min of peak oxygen consumption or 35% of ppoVO_2_ max are not candidates for lung resection, and chemo or radiotherapy might be other options (19, 30). This preoperative evaluation modality is summarized in Figure 1.

### How to improve functional status?

Chosen exercise therapy may be adapted according to the surgical disciplines such as colorectal (31, 32), orthopedic (33), cardiac (34), thoracic (27) and non-cardiac (35) surgeries. Generally, two major aerobic exercise concepts may be chosen as a classic rehabilitation modality to ameliorate aerobic capacity: Endurance training (ET) or High-intensity interval training (HIIT). Either way, increased cardiac output, efficient oxygen extraction, and enhanced muscle contractility is expected with exercises (36). ET is the most preferred approach with intention to provide long-lasting and gradual improvement by training at 40–60% intensity of maximal effort which is accepted as “moderate,” and generally lasts 6–12 weeks. On the other hand, HIIT aims bigger improvements in a short time period (generally shorter than 4 weeks) with a 60–80% of maximal effort training intensity (37–39). Usually, incline walking, running, cycling or rowing are suitable as aerobic exercises, and for that, ergometric cycle, treadmill or rowing ergometer may be used. Activities like walking and cycling may be chosen for “moderate” intensity activities since these do not require skills. For high-intensity activities, running, rowing, and spinning are well-suited exercise types. Heart rate (HR) is the indicator for achieving desired exercise intensity, and to determine the target heart rate, “Heart Rate Reserve” method is used. It is explained with the formula below (40).

$\begin{array}{l} \mathrm{Target}\;\mathrm{Heart}\;\mathrm{Rate}=\\ {}[(\mathrm{Maximal}\;HR-\mathrm{Resting}\;HR]\;x\%\mathrm{intensity}]+\\ \;\mathrm{Resting}\;HR\text{.} \end{array}$

Maximal HR is generally calculated by substracting age from 220, which gives an approximate value.

HIIT is generally performed by doing repetitive short duration of vigorous effort which was initially described by Tabata et al. as 7–8 sets of 20 s exercise and 10 s of rest between the sets (41). This concept can be adapted for different exercise types including rowing, spinning, and running, and session durations change accordingly which generally does not exceed several minutes (42, 43). However, ET is a relatively more continuous modality that takes around 30–50 min spending moderate effort (40).

In a study, which examines moderate continuous training versus HIIT, by Villelabeitia-Jaureguizar et al., an explicit improvement in peak oxygen consumption and heart rate recovery (HRR) were observed with supervised, hospital-based HIIT training, even in the acute period of post-revascularization (38). Accordingly, HIIT has provided better improvement in HRR, which is accepted as a predictor of cardiovascular mortality both in healthy and patients with coronary heart disease (CHD), yet vast majority of the patients benefited from the intervention in terms of increasing VO_2_ max 3 to 5 mL/kg/min (38). Of note, many studies demonstrated significantly more increment of peak oxygen consumption with high-intensity training in comparison to moderate continuous exercises which refers to classical endurance training (44–47).

Recent literature has also focused on the efficacy of preoperative exercise and rehabilitation on the incidence of PPCs after thoracic surgery. However, the results were not as expected. Licker et al. demonstrated a good increase in VO_2_max (mean value of 2.9 mL/kg/min) and 6-min walking test (6MWT) with short-term HIIT, and yet it did not reduce the occurrence of PPCs (47).

Inspiratory muscle training (IMT) is also proposed in the current literature, which is based on identical hypothesis of aerobic exercises regarding improved strength and endurance of the inspiratory muscles. Brocki et al. have demonstrated a better physical activity in high-risk patients who received two-week IMT before lung resection surgeries. We should underline that these patients were either above 70 years of age or had FEV_1_ and/or FVC lower than 70% (48). Similar results were also observed in another trial which included lung transplantation candidates (49).

### Understanding respiratory muscles

Skeletal muscle fibers have four isotypes of myosin heavy chains which are Slow 1, Fast 2A, Fast 2B, and Fast 2X. As a physical property, slow type fibers represent high oxidative metabolic capacity which makes them more resistant to fatigue, and the fast type fibers are considered easily fatigable (except Fast 2A, which shows intermediate resistance to fatigue). Typically, diaphragm has relatively small fiber size, abundant capillaries, and high oxidative capacity that provides fatigue resistant structure allowing continuous respiration activity without exhaustion. About 50% of the fibers have slow myosin and 40% is myosin Fast 2A in the diaphragm. However, this proportion may adapt or change according to different pathological conditions (50). Accessory respiratory muscles become more important in case of a shift from quiet breathing to tachypnea, and as expected sternocleidomastoid, scalenes, and intercostal muscles represent a higher percentage of fast fibers (51).

Sedantary lifestyle and immobilization are associated with a conversion from slow to fast fibers indicating a serious drop in oxidative capacity (52). Acute postoperative period generally requires the use of accessory muscles for a proper recovery from the residual effect of mechanical ventilation under full neuro-muscular block. Therefore, having “fit” inspiratory muscles might be the key for a better oxygenation and less complications. Studies indicate a well transformation from myosin 2X to 2A fibers with either endurance or high-intensity training, which is the logic behind adding respiratory training into prehabilitation concept (53, 54).

### Current concepts of prehabilitation and integration of IMT

Literature represents many clinical trials and several meta-analyses documenting the possible benefits of prehabilitation modalities without respiratory exercises in different fields of surgery (18, 55–57). General idea is that prehabilitation is associated with reduced length of stay and postoperative complications (some studies declare a reduction in PPCs also), and better physical status. However, the major problem is the heterogeneity of the study designs with really small sample sizes, which lowers the quality of evidence (57).

Current trends in prehabilitation cover both aerobic and respiratory training. Inspiratory muscle training (IMT) is the most preferred one among the respiratory exercises, and appears to be superior to expiratory exercises in terms of improving pulmonary functions such as FEV_1_ and FVC (58).

Most clinical trials designed as “preoperative” intervention have chosen “endurance” training concept with resistive-flow devices, which allows adjusting a workload for IMT. For that, a maximum inspiratory pressure (MIP) was measured prior to training and often 50–60% of MIP was set as initial workload. Training sessions may be completed this way or else, by increasing the workload 10% of MIP weekly (59).

Many investigations have demonstrated a significant amelioration in pulmonary functional parameters, aerobic capacity and oxygenation with IMT. In a meta-analysis by Gomes Neto et al., patients (*N* = 386) who received IMT before undergoing “cardiac” surgery have exhibited reduced length of stay (by 2 days), increased inspiratory pressure (by 16 cm H_2_O), increased FEV_1_ and FVC (3 and 4.6%, respectively), and most importantly reduced pulmonary complications (by a risk ratio of 0.6) (60). In another meta-analysis (*N* = 295), risk of PPCs is shown to be even halved (RR: 0.48) when a composite of upper abdominal and cardiothoracic surgeries was evaluated (61). In another meta-analysis which included 12 clinical trials by Kendall et al., relative risk ratio regarding the PPCs for patients receiving preoperative IMT was found to be 0.50, also (59). Moreover, a Cochrane analysis has concluded specifically reduced length of stay, pneumonia, and atelectasis with preoperative IMT in cardiac and major abdominal surgeries, yet its effect on all-cause mortality is uncertain (62).

Latest randomized clinical trials (RCTs) support the idea of IMT lowering PPCs in lung resection surgeries (63–66). A recent, relatively small sample size study by Huang et al. have demonstrated favorable outcomes with a “perioperative” training modality which started 3 weeks before the surgeries and continued up to postoperative 4 weeks, and accordingly PPC occurrence was lower in IMT group (63). Thoracic surgery represents considerable importance since PPC incidence is observed more than any other surgical group, and more data regarding the postoperative outcomes of sole lung resections are needed.

Existing literature are quite heterogeneous in terms of study design and intervention modalities which makes it difficult to elucidate “ideal” prehabilitation. Type of intervention (unimodal or multimodal), type of exercise (ET only, HIIT only, IMT only, IMT + HIIT, or IMT + ET), and acceptable duration of intervention are the main points that need clarifying. In one of the latest meta-analyses, which investigated non-small cell lung cancer resection studies, postoperative complications including PPCs were shown to be reduced by 30% with preoperative exercise training (67). However, one should not miss-out, the biggest sample sized study was with 151 patients in which the preoperative intervention was HIIT only. Seemingly, exercise capacity, pulmonary functions, and quality of life improves with preoperative exercise training, yet more definitive data regarding the duration, intensity, and training method are needed to adapt prehabilitation into routine clinical practice (18).

## Discussion

### Controversies regarding prehabilitation modalities

Malignancy surgeries generally necessitate a fast-track preparation, and both patients and surgeons may not desire long awaiting process. Therefore, duration of prehabilitation is one major determinant to define, in order to provide most benefit in a certain amount of time. Literature represents a general understanding which is simply longer the training duration (mostly longer than 4 weeks) better the outcomes (27, 65, 68–70). However, Benzo et al. observed “feasible” success with short-term prehabilitation (total 10 sessions in 1 week) with reduced length of stay and less prolonged chest tube drainage after thoracic surgeries (64). Moreover, Lai et al. have managed to demonstrate a significant decrease of PPCs (13.3% in control group vs. 36.7% in IMT + ET group, *p* = 0.037) in patients undergoing lung cancer surgery (71). On the contrary, Licker et al. have failed to show such improvements with HIIT-only prehabilitation modality (47). Therefore “inspiratory” training might be the major determinant here.

Another question mark is onto the superiority of one aerobic training over another. The number of accordingly designed studies is quite limited. HIIT is generally difficult to adapt into daily routine because of its highly competitive nature and requirement of professional supervision. In order to compare the effects of aerobic exercises on surgical outcomes, one study by Van Adrichem et al. have an appropriate design testing IMT + HIIT versus IMT + ET which was conducted in esophagectomy patients, and PPCs were significantly reduced in IMT + HIIT group (72). Daily personalized supervision in hospital settings require advanced facilities and additional cost that is considered unfeasible for routine clinical practice. At this point, endurance exercises may be chosen for aerobic training since it is more suitable for remote supervision and may not require one-to-one interaction between the healthcare givers and the patients. Home-based supervision concept has shown to be presenting favorable results in obtaining better physical activity, functional status, and occasional improvement in postoperative complications (49, 66). Of note, existing home-based therapy related studies reflect heterogeneous data that underpower the meta-analyses (16). Developed technology has made it possible to use communication devices more effectively (73, 74). During the Covid-19 pandemic, physicians were forced to generate alternative methods such as telemedicine which helped them to reach the patients and structure appropriate rehabilitation programs (75).

### “Multimodality” in prehabilitation

**Figure 2:**
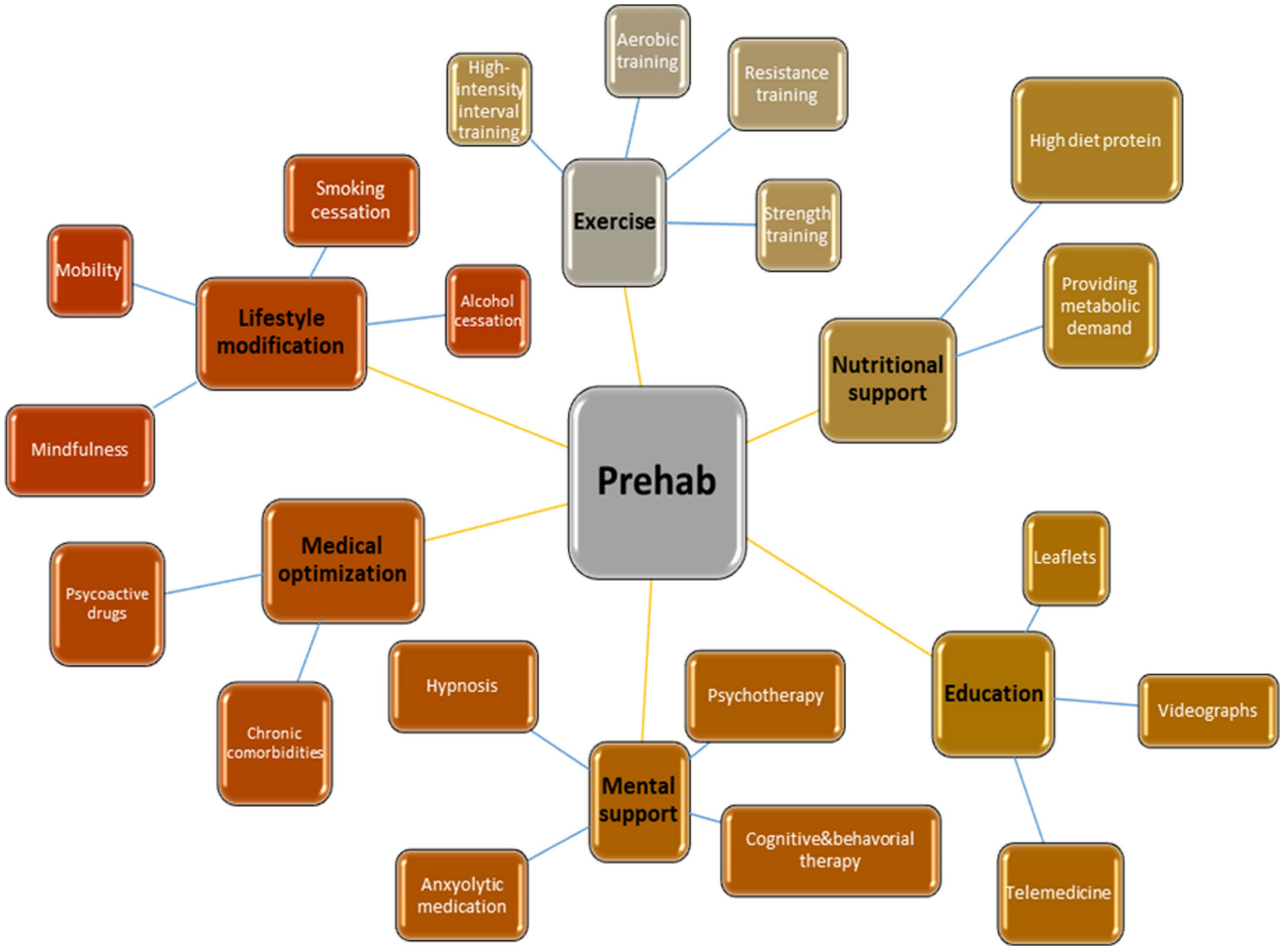
Multimodal approach of prehabilitation.

**Figure 3:**
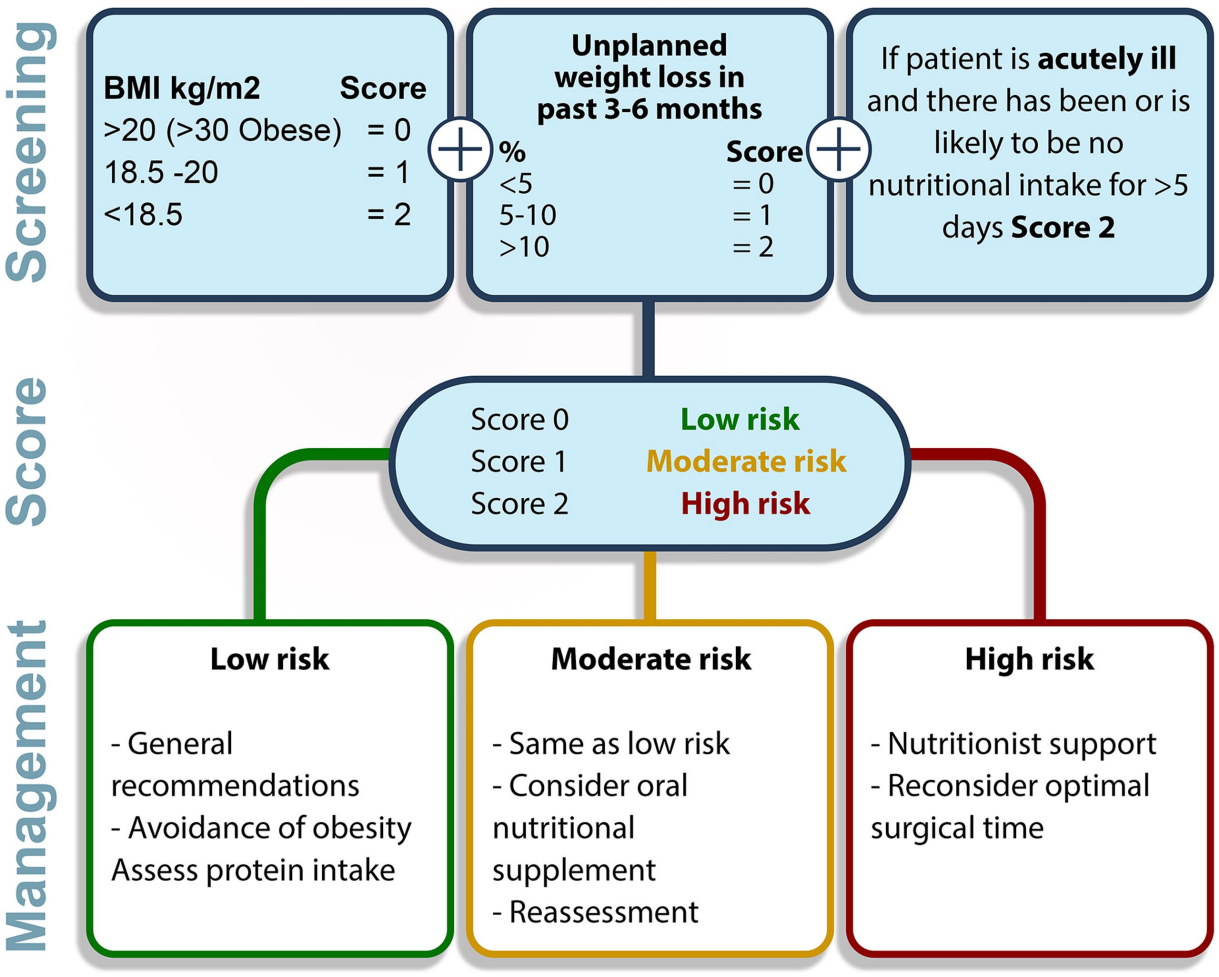
Malnutrition universal screening tool (MUST) (77, 78).

Effective prehabilitation demands multimodal approach which includes additional concepts of nutrition, psycho-cognitive support, education, lifestyle change, and medical optimization (Figure 2). “Nutrition” stands as an important modifiable risk factor since it is known that malnutrition is related to loss of muscle mass and impaired immunity. Currently, evaluation of the nutrition status is recommended by the ERAS program (76). Oral supplements should be considered in case of severe malnourishment which also requires multidisciplinary approach to decide ideal timing and nutrition program, and this should be based on individuals’ requirements since waiting for such intervention might delay surgery and change the surgical outcomes. If current prehabilitation program includes exercise training, 1.5 mg/kg high-quality protein supplementation should be provided since oral dietary might not contain such high amounts of protein (77). To identify malnourishment in adults, Malnutrition Universal Screening Tool (MUST) is a practical flowchart and may be chosen preoperatively (Figure 3) (77, 78).

One other aspect of prehabilitation is “psycho-cognitive support” which shows extreme importance in elderly population. In order to guide the therapy, the Hospital Anxiety and Depression Scale (HADS) may be used, and several approaches (Cognitive-behavioral therapy, mindfulness, deep breathing, meditation) may be chosen to reduce anxiety (79, 80). A strong understanding of the perioperative period by the patient also is an important entity to reduce fear, discomfort, and pain. Visual materials such as leaflets or videos may be prepared to briefly inform the patients. Cessation of smoking and alcohol consumption should be promoted. Despite the controversy regarding the duration of smoking cessation, guidelines recommend quit smoking as soon as the surgery decision is made. Continued smoking is associated with poor postoperative outcomes (76, 81). Generally speaking, a 4-week cessation of smoking and alcohol normalize immune responses and reduces the surgical complications, however one might consider whether this amount of time may deteriorate malignity progression (76, 77).

### Integration to ERAS programs

“Enhanced Recovery After Surgery” (“ERAS”) has become a-literally- trademark in recent years. Starting in gastrointestinal surgery, recommendations to improve the ERAS approach for numerous different operations have been published, mostly by ERAS® -society. In PubMed, the search for a combination of “ERAS AND prehabilitation” reveals 126 results.

On one hand, prehabilitation appears to be a natural part of ERAS; it is not surprising that both concepts improve in parallel ways. On the other hand, among these 126 results, some of them report the difficulties, limitations, and uncertainties of prehabilitation, which are partly described in this paper. Interestingly, there are also uncertainties between different ERAS-protocols: Single-center protocols deviate from each other and from the one of the ERAS®-society.

In different ERAS protocols for different types of operations, researchers have focused –among others- on the intraoperative interventions such as fluid management or ventilation modalities for the last two decades (82–84), but ERAS includes also suggestions for pre- and postoperative approach, with prehabilitation as a preoperative one.

As a still evolving entity, multimodal prehabilitation shows promising features in terms of reducing morbidity in the perioperative period with a focus on PPC. Existing data are quite heterogenous, however, a great effect may be observed in the future as the concept is improved. Duration of prehabilitation is one big controversy, and more clinical data are needed to gain better understanding. Yet, one might suggest improving aerobic capacity with short-term exercises may ameliorate perioperative outcomes. Therefore, it would be best to develop a personalized program for every individual which requires a well organization and cooperation between every related discipline that includes anesthesiologist, surgeon, physiotherapist, psychologist, and nutritionist.

Implementing a 2-week schedule with aerobic endurance and inspiratory muscle training exercises along with appropriate nutrition and lifestyle changes would be the answer for “How to prehabilitate?” question. Perhaps, looking for better answers while trying to shorten the duration could be more beneficial on the surgery side in order to not miss out actual “surgically treatable” window. Apparent studies show that the focus will be on home-based and individualized interventions in the future, and hopefully will reflect more favorable results.

## Author contributions

All authors listed have made a substantial, direct, and intellectual contribution to the work and approved it for publication.
